# Editorial: Quantum Dots for Biological Applications

**DOI:** 10.3389/fbioe.2022.930213

**Published:** 2022-05-16

**Authors:** Sameer Hussain, Gopinath Packirisamy, Krishna Misra, Mohammad Tariq, Md Palashuddin Sk

**Affiliations:** ^1^ School of Chemistry, Xi’an Jiaotong University, Xi’an, China; ^2^ Nanobiotechnology Laboratory, Department of Biosciences and Bioengineering, Indian Institute of Technology Roorkee, Roorkee, India; ^3^ Department of Applied Science, Indian Institute of Information Technology, Allahabad, India; ^4^ LAQV, REQUIMTE, Departamento de Química, Faculdade de Ciências e Tecnologia, Universidade NOVA de Lisboa, Caparica, Portugal; ^5^ Department of Chemistry, Aligarh Muslim University, Aligarh, India

**Keywords:** quantum dots, Cdots, SQDs, biofilm, biosensor, bioimaging

Quantum dots (QDs) are semiconductor nanoparticles with the sizes of a few nanometers (2–10 nm) and represents a revolutionary class of light-emitting nanomaterial, which has promising applications in the diverse research areas ranging from optoelectronics to theranostics. The properties of QDs are usually different from larger particles due to quantum confinement effects. Owing to their unique and exciting properties, such as excellent photostability, narrow emission band with a wide range of spectral wavelengths, and high photoluminescence quantum yield, QDs have emerged as a preferred photoluminescent material over conventional organic fluorophores for numerous biological and biomedical applications. Full color imaging with QDs is possible due to their size dependent emission property.

The two most common techniques for the synthesis of QDs include the top-down method and the bottom-up approach. Moreover, QDs can be functionalized with appropriate ligands called receptors for targeting purposes. Recent years have witnessed a variety of QDs with potential applications in imaging, tracking, sensing, drug delivery, and phototherapy. Biologists experimenting with living cell composites generally use QDs to repair damaged neuralpathways or deliver drugs. However, the toxicity of most QDs still remains the biggest challenge for scientists working in related areas. Thus, the development of non-toxic, biocompatible, and bright QDs with viability in biological systems is one of the sought-after avenues of research.

This Research Topic is a humble effort to promote and gather newer developments related to the preparation and biological applications of QDs. Researchers were invited to contribute to novel synthetic methodologies of QDs especially sustainable techniques and cover their applications in the realms of biology. Overall, we have collected two original research articles, one review paper, and one minireview, which focused on the preparation of QDs for sensing, imaging, diagnosis, antibacterial therapy and provide an update on emerging trends of QDs. The following section briefly elaborates on the articles and review papers collected on this Research Topic.

Visible light-activated QDs with inhibitory properties against harmful microorganisms could be highly beneficial for preventing the formation of microbial biofilms on various surfaces. Yang et al. *,* in their work, developed 2,2′-(ethylenedioxy)bis (ethylamine) capped photoactive carbon dots (Cdots) by simple amidation reaction and demonstrated their inhibitory effect against the formation of *B. subtilis* biofilm as well as the inactivation of *B. subtilis* cells inside the biofilm. Moreover, improved photo-induced inactivation of biofilm-associated cells was observed when Cdots were strategically coupled with chelating agent ethylenediaminetetraacetic acid (EDTA). Thus, the combination of visible light, Cdots, and EDTA provides an excellent antibacterial platform for preventing and eradicating biofilms, a long-standing problem in the biomedical field and industries.

In their exciting work, Li et al. reported the preparation of fluorescent and less toxic peptide modified carbon polymer dots (pep-CPDs) to detect protein S100-β, which acts as a molecular biomarker for early diagnosis of mild traumatic brain injury (mTBI). mTBI is a type of traumatic brain injury that is difficult to diagnose precisely in the early stage due to the absence of reliable biomarkers and suitable detection methods. In contrast to conventional detection methods, the pep-CPDs-based system is found to be rapid, sensitive, less toxic, and trustworthy towards the detection of biomarker S100-β, facilitating efficient diagnosis of mTBI. Moreover, the pep-CPDs displayed excellent fluorescence imaging performance both *in vitro* and *in vivo,* as evidenced by imaging of S100-β inside the HeLa cells and C57 mice which indicated the potential of the system in clinical and translational applications.

Parmar’s et al., in their review paper, elegantly and comprehensively described the common synthetic procedures and fundamental properties of Cdots derived from natural sources such as biomolecules and medicinal plants. Special attention has been paid in highlighting the theranostic applications of such Cdots through cell imaging, drug delivery, and phototherapy considering cancer as the model disease. Moreover, the authors have also shed light on the current scenario and future perspectives of Cdots-based nanoprobes for clinical applications.

The minireview published by Zhou et al. on this Research Topic magnificently highlights the synthesis of sulfur quantum dots (SQDs) mainly for biosensing and imaging applications. SQDs have recently emerged as a new candidate in the domain of non-metallic luminescent QDs and exhibit several exciting features such as facile synthetic procedure, low toxicity, ultra-small size, excitation tunable fluorescence, unique composition, etc., which facilitates their applications in biology. Thus, a variety of SQDs has been discussed that have been employed for potential biological applications such as cell and tissue imaging, detection of biomolecules, metal ions, temperature, etc. Finally, the current challenges and future outlook of SQDs in the arena of bioimaging and biosensing are also discussed briefly.

In conclusion, this Research Topic provides state-of-the-art advances in low toxic (non-toxic) QDs such as Cdots, pep-CPDs, and SQDs for various biological applications, including bacterial inactivation, sensing, bioimaging, and therapy. The review papers collected on this Research Topic also revealed that regardless of the significant advancements in the related area, low toxicity and poor biocompatibility of most QDs continued as the biggest challenge for the biomedical scientists because it restricts their applications in clinical biology. Moreover, the inability of many QDs to cross the blood-brain barrier greatly hinders their application in targeting brain tumors. Besides, the cost-effective synthesis and industrial-scale production of QDs are also difficult tasks that are required to be overcome in the near future. Future studies necessitate the development of QDs with good biocompatibility, low toxicity, facile synthetic methods, and cell-penetration ability.

